# An elderly female with adult-onset Still’s disease initially misdiagnosed as prosthetic joint infection after total knee arthroplasty: lessons in differential diagnosis and treatment

**DOI:** 10.1186/s12877-020-01925-w

**Published:** 2020-11-27

**Authors:** Xufeng Jiao, Zheng Li, Shuai An, Jiang Huang, Guangzhong Yang, Yi Zhao, Jianghua Shen, Yanqi Chu, Charlie C. Yang, Guanglei Cao

**Affiliations:** 1 Department of Orthopedics, Xuanwu Hospital Capital Medical University, 45 Changchun Street, Xicheng District, Beijing, 100053 China; 2 Department of Rheumatology and Immunology, Xuanwu Hospital Capital Medical University, 45 Changchun Street, Xicheng District, Beijing, 100053 China; 3 Department of Pharmacy, Xuanwu Hospital Capital Medical University, 45 Changchun Street, Xicheng District, Beijing, 100053 China; 4 Colorado Joint Replacement, 2535 S Downing Street, Suite 100, Denver, CO 80210 USA

**Keywords:** Adult-onset Still’s disease, Prosthetic joint infection, total knee arthroplasty, Knee revision, Diagnostic treatment

## Abstract

**Background:**

High fever, knee swelling and pain after knee arthroplasty are often considered as symptoms of acute prosthetic joint infection. However, similar symptoms can also present as primary manifestations of adult-onset Still’s disease, which creates some interference in differential diagnosis. To our knowledge, this is the first published case of misdiagnosis of adult Still’s disease after total knee arthroplasty, who was initially misdiagnosed as an prosthetic joint infection due to the above-mentioned symptoms. The symptoms of the knee infection was not relieve after several revisions and continous antibiotic treatment. Finally, after several consultations and repeated evaluation it was diagnosed as adult-onset Still’s disease.

**Case presentation:**

A 77-year-old female who underwent bilateral total knee arthroplasty 6 years ago was admitted to our hospital with high fever, right knee effusion and painful knee. Based on the results of joint fluid aspiration and culture, we treated the right knee as acute hematogenous prosthetic joint infection. After three debridement and revision surgeries, the patient’s symptoms continued to persist. Subsequent manifestations of other symptoms such as typical rash and sore throat and laboratory examination suggested the possibility of adult-onset Still’s disease. So she underwent diagnostic steroid hormone therapy at the recommendation of a rheumatologist, and a final revision was performed after symptom was controlled. At the one-year follow-up, the patient’s symptoms completely resolved and the knee revision was functioning well.

**Conclusions:**

When joint swelling and pain occurs after knee arthroplasty, the possibility of joint infection should not only be considered, but rheumatic autoimmune diseases should also be differentiated. Because the manifestations of joint infection and rheumatic immune disease sometimes overlap highly, when reasonable treatment over a period of time fails to relieve symptoms and signs, we should notice subtle differences in symptoms and laborotary tests and look for other diagnostic possibilities in time.

## Background

Adult-onset Still’s disease (AOSD) is a systemic inflammatory disease of unknown etiology [1]. Spiking fever, arthritis, and maculo-papular salmon-pink evanescent rash are the most common symptoms [2]. In addition, there can be sore throat, hepatosplenomegaly, enlarged cervical lymph nodes and pericarditis [2]. AOSD can lead to leukocytosis, increased neutrophil percentage, thrombocytopenia, inflammatory anemia, increased erythrocyte sedimentation rate (ESR) and C-reactive protein (CRP) level [3], mild to moderate abnormalities in blood coagulation, and abnormal liver function [4]. Patient’s serum ferritin level can be significantly high, and glycosylated ferritin level can be reduced to ≤20% [5, 6]. While AOSD is a clinical diagnosis, there is no definite diagnostic examination. At present, the Yamaguchi criteria is often used for clinical diagnosis [7].

Fever, joint symptoms and erythema are also important diagnostic bases for prosthetic joint infection (PJI) after total knee arthroplasty (TKA) [8]. Laboratory tests can also show inflammation such as leukocytosis, increased ESR and CRP levels. These symptoms may overlap somewhat with the diagnosis of AOSD.

We searched “knee/hip arthroplasty & AOSD” and “total knee/hip arthroplasty & AOSD” in several databases such as Pubmed, but did not find relevant cases, especially related reports that were misdiagnosed as PJI. To our knowledge, the development of AOSD after total knee arthroplasty has never been reported to date. The purpose of this case report is to provide additional knowledge on suspected infection after knee arthroplasty. Because cognitive impairment due to Alzheimer’s disease, written informed consent for publication of our patient’s clinical details and clinical images was obtained from the guardian relative of the patient.

## Case presentation

A 77-year-old Chinese female was admitted to hospital with high-grade fever, sore throat, and swelling of the right knee. The intermittent high fever(> 40 °C), which started 20 days ago, had no obvious cause,and was accompanied by chills, redness, pain and swelling of the left ankle. The severe and unbearable pain often occurred during fever, and was slightly relieved after the fever subsided. However, a few days later, these syptoms disappeared while the right knee showed the same symptoms. The patient initially underwent bilateral TKA six years ago and recovered uneventfully and had well functioning TKAs. She also had a history of Alzheimer’s disease.

Her initial body temperature was 39.2 °C in the evening of admission. The right knee was swollen, and she experienced pain with tenderness. The floating patellar test was positive on the right knee; and negative on the left. The range of motion was 0–100° for the right knee and 0–120° for the left. There was no obvious instability in either knee joint.

Laboratory examination results are shown in Table 1. The abdominal ultrasound showed no signs of hepatosplenomegaly and the X-ray showed no obvious abnormality in both knee joints and chest.

**Table 1:** Laboratory examination results during two hospitalizations

Component	First admission	Second admission	Before discharge	Reference range
WBC (× 10^9^/L)	14.49	8.11	7.24	4–10
Neutrophils (%)	85.5	65.7	5.04	50–75
Hemoglobin (g/L)	99	124	106	110–150
Platelet (×10^9^/L)	252	217	217	100–300
CRP (mg/L)	73.6	1.68	2.44	1–8
ESR (mm/hr)	36	5	7	0–20
Total protein (g/L)	58.20	58.61	51.83	60–80
Albumin (g/L)	26.42	34.32	30.28	35–55
ALT (IU/L)	63	26	18	5–40
AST (IU/L)	105	27	25	8–40
Bilirubin, direct (umol/L)	1.98	1.93	2.57	0–8.24
Bilirubin, total (umol/L)	4.30	5.46	7.80	3.42–15.1
LDH (IU/L)	658	275	not done	109–245
INR	1.24	1.04	not done	0.8–1.2
PT (sec)	15.4	13.5	not done	11–15
APTT (sec)	43.1	31.5	not done	25–43.5
D-Dimer (ug/mL)	10.65	2.17	not done	0.01–0.5
G experiment (pg/mL)	< 37.5	not done	not done	0–70
GM experiment (S/CO)	0.2	not done	not done	0–0.5
Ferritin (ng/mL)	(after the consultation) > 1500	280.3	not done	11–306
ANA	(after the consultation) negative	negative	not done	negative
RF	(after the consultation) negative	negative	not done	negative
ASO titer (IU/ml)	not done	26.2	not done	26–116

*ALT* alanine amino transaminase, *APTT* activated partial thromboplastin time, *ANA* antinuclear antibody, *ASO* antistreptolysin O, *AST* aspartate aminotransferase, *CRP* C-reactive protein, *ESR* erythrocyte sedimentation rate, *G test* 1,3-β-D-glucan test, *GM test* galactomannan test, *INR* international normalized ratio, *LDH* lactic dehydroginase level, *PT* prothrombin time, *RF* rheumatoid factor, *WBC* white blood cell

**Fig. 1:**
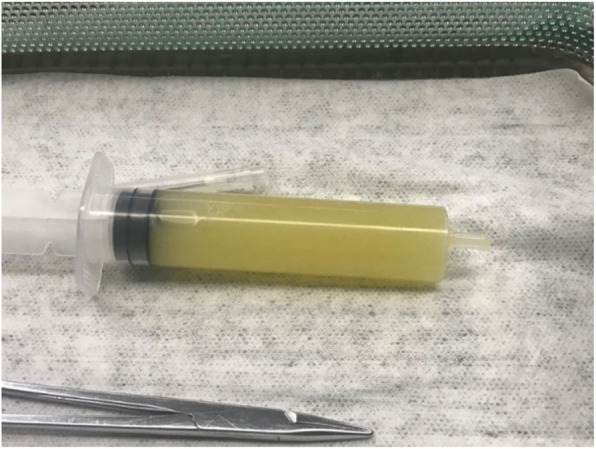
The patient’s right knee aspiration fluid on the day of firtst admission showed a yellowish-green turbid character

The right knee was aspirated upon admission, and the aspiration fluid showed a yellowish-green turbid character (Fig. 1). Routine examination of the fluid showed inflammatory characteristics (Table 2). No fungi or bacteria were found in the aspiration fluid. Venous blood culture was negative. Thereafter, blood culture was carried out every time the temperature increased significantly (a total of five times), and the results were all negative. After consultation, the pharmacy department provided diagnoses of erysipelas and knee arthritis, and treated the patient with penicillin. However, the patient’s body temperature still fluctuated above 38 °C over the next two days, so treatment was changed to vancomycin and levofloxacin.

**Table 2:** Results of several joint fluid analysis

Side	1	2	3		4		5
	right	right	right	left	right	left	right
Color	pale yellow	–	bloody	deep yellow	–	–	–
Pellucidity	slightly turbid	–	turbid	turbid	–	–	–
Total cellular score(× 10^6^/L)	46,006	–	193,259	41,608	–	–	–
White blood cell count(×10^6^/L)	37,006	–	17,259	26,808	–	–	–
Mononuclear leucocyte	4.2%	–	9%	7%	–	–	–
Multinuclear leucocyte	95.8%	–	91%	93%	–	–	–
Protein (mg/dL)	–	381	–	–	–	–	–
Glucose (mmol/L)	–	0.94	–	–	–	–	–
Chlorine (mmol/L)	–	89	–	–	–	–	–
Culture	negative	*Staphylococcus epidermidis*	negative	negative	negative	human staphylococci and staphylococcus cohnii	negative

1:On the day of first admission; 2: During right knee DAIR; 3:After DAIR; 4: During left knee DAIR and placing the antibiotic bone cement spacer in the right knee; 5: During removing right knee bone cement spacer

Considering the patient’s symptoms and test results, acute PJI could not be ruled out. We performed debridement, antibiotics, implant retention (DAIR) for the right knee five days after admission. The grinding tissue fluid was taken for culture and biochemical examination (Table 2).

The symptoms and laboratory results were slightly improved postoperatively. But on the fourth day after the operation, the patient’s temperature spiked again, reaching over 39.0 °C. A punctate congestive rash was distributed symmetrically over the neck and trunk. At the same time, the patient developed a cough, expectoration, and wheezing. The leukocytes level and the percentage of neutrophils increased significantly. CT scan showed bilateral lung infection with pleural effusion. It also showed enlargement of mediastinal and bilateral axillary lymph nodes. Echocardiography showed a small amount of pericardial effusion and decreased left ventricular diastolic function. We treated it as pulmonary infection, cardiac insufficiency and drug-borne allergy. The previous antibiotics were replaced with linezolid cefoperazone/sulbactam and furosemide, followed by an intravenous drip of dexamethasone 10 mg for three days. During the use of dexamethasone, the patient’s temperature was normal. The respiratory symptoms improved significantly, and the rash gradually subsided. However, her temperature rose above 37.5 °C again after stopping dexamethasone. The left knee displayed redness, swelling, and pain, and the floating patellar test was positive. Both 1,3-β-D-glucan test (G test) and galactomannan test (GM test) were negative, and the sputum culture showed presence of *acinetobacter pittii*. Treated with an intravenous infusion of imipenem, linezolid, and fluconazole, the patient reported that pain in both knees and the rash intensified. The aspiration fluid collected from both knees showed no bacteria in the culture but the possibility of suppurative arthritis once again (Table 2).

Considering that the infection remained uncontrolled, we removed the right prosthesis again, placed the antibiotic bone cement spacer, and performed DAIR for the left knee. We took synovial grinding tissue fluid from both knees for culture, and found Human staphylococci and Staphylococcus cohnii in the left. The temperature remained abnormal postoperatively and even gradually increased over the next few days, peaking around 14:00 each day. Two weeks later, the patient’s right knee swelled again with sticky secretions, but the culture of the joint aspiration fluid was negative. Subsequent mycoplasma pneumoniae and tuberculosis antibody tests were negative. T-SPOT test was positive, so oral isoniazid was given for treatment. A few days later, the right knee’s symptoms worsened, and a rash appeared.

**Fig. 2:**
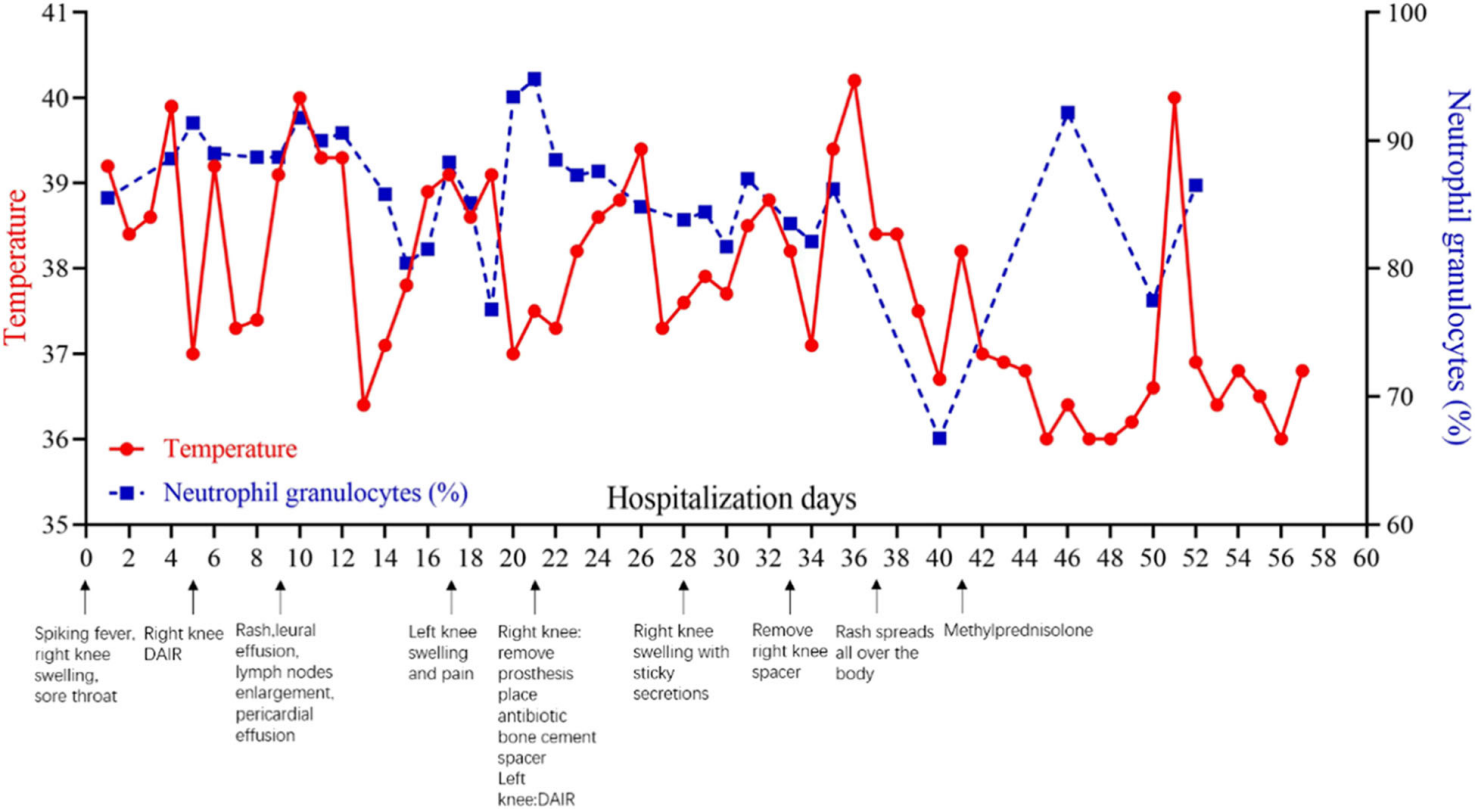
Body temperature, neutrophils, important symptoms, and treatment timeline during the first hospitalization

Then, the spacer in the right knee was removed, and the knee was fixed by bracing. However, the result of articular fluid analysis remained negative. On the third day after operation, the rash had spread all over the body, and the temperature rose again. After four operations on both knees, the fever and rash got worse and worse, so we thought it might not be a simple PJI and consulted the rheumatic immunology department. After excluding the possibilities of malignancies and hematological diseases, they found the patient’s symptoms matched those of AOSD. According to the Yamaguchi criteria [7], our patient had four major features (spiking fever, arthralgia, rash, and leukocytosis) and four minor features (sore throat, abnormal liver function, lymph nodes enlargement and negative ANA/RF). Using a combination with antibiotics, we continued to prescribe intravenous infusion of methylprednisolone powder 40 mg once a day for three days, then changed the medication to oral prednisone 50 mg. After treatment, the patient’s temperature returned to normal. Both the rash and symptoms of bilateral knees disappeared. A week later, the CRP level decreased to normal (5.06 mg/L) while ESR level was slightly higher (30 mm/h). The patient was discharged. A brief diagram of the patient’s disease changes is shown in Fig. 2.

**Fig. 3:**
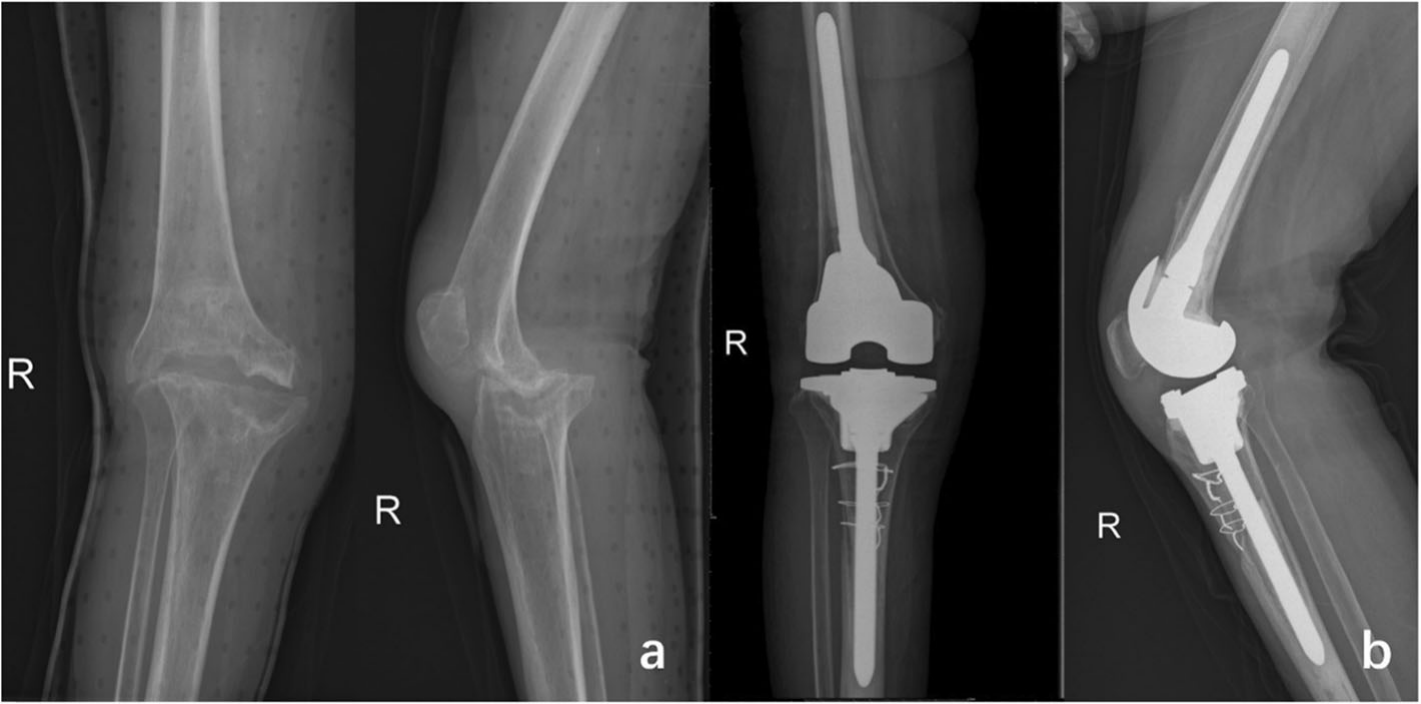
a-After the right knee spacer was removed for 4 months, the X-ray film showed obvious bone defects and reduced space in the distal femur and tibial plateau; b- During the second hospitalization, the knee joint was reconstructed with ACCK prosthesis (ACCK Knee Prosthesis; AK; Beijing, China)

After discharge, the patient’s right lower limb was fixed with a brace, and she continued taking prednisone 50 mg once a day. The symptoms were well-controlled, and the medication dose decreased gradually after a month. The patient was re-admitted four months later and was re-examined (Table 1). The X-ray of the knee showed bone defects and reduced space in the distal femur and tibial plateau (Fig. 3). We performed a revision of the right knee with ACCK prosthesis (ACCK Knee Prosthesis; AK; Beijing, China).

At one year follow-up after the operation, her right knee flexion was 100° with 15° extension lag and the HSS score was 76. The patient had discontinued prednisone for one month without any systemic or local symptoms.

## Discussion and conclusion

Bywater first proposed AOSD in 1971 [9], but since its characterization, the development of AOSD after arthroplasty has not been reported. To our knowledge, this is the first case of AOSD developed after TKA in the world.

AOSD usually affects young people [10], with bimodal peaks at ages 15–25 and 36–46 years [10], but the 77-year-old female in our report is far beyond the expected age.

Triad of symptoms - spiking fever, arthritis or arthralgia, and salmon-pink transient maculopapules - are common features of AOSD [10]. Arthritis mainly involves the knee, ankle, wrist, and proximal interphalangeal joint [4]. Among them, the knee joint is the most often affected [2], for patients with a history of knee surgery, this feature can make the diagnosis difficult.

Looking back at the entire diagnosis and treatment process, we overlooked some signs that have suggested the diagnosis was not PJI:

1. The patient had cognitive impairment caused by Alzheimer’s disease, so her complaint of ankle swelling, which had subsided on admission, was ignored by the doctor. The symptom of multi-joint involvement often indicates a systemic disease rather than a joint infection, which is also in line with the characteristics of AOSD [4].
2. We considered that PJI was induced by hematogenous infection and performed DAIR after her admission. This is because the patient had a sudden persistent high fever with sore throat, whereas simple knee infections often have local symptoms with mild to moderately elevated body temperature. Paradoxically, a series of imaging examinations did not reveal any other primary focus of infection. This neglected result also indirectly negated the diagnosis of hematogenous PJI.
3. We applied the most potent antibiotics after the right knee DAIR. Even so, the high fever did not abate and the symptoms even progressed to the left knee. This indicated again that it was not due to infection. Because if it were PJI, after debridement - even if the debridement was incomplete - the condition would have improved after antibiotics were applied. Meanwhile, the patient developed a rash, which we interpreted as an allergic rash caused by vancomycin at the time. We did not notice that it became more congested in high fever periods and relieved in intermittent periods, which happens to be highly suggestive of AOSD.
4. When the patient developed pneumonia, pericarditis and enlarged lymph nodes after the operation, we thought that a hematogenous infection could be the reason at that time. However, five blood cultures performed during the high fever were all negative, which once again suggested that the possibility of infection was minimal. We even conducted a bacterial genetic test of the joint fluid, and the result was also negative. Infection is a huge consumption process for the aged, yet this patient did not show any weakness between febrile episodes. All these signs suggested that we should consider other diagnoses rather than PJI.
5. When many targeted treatments did not achieve the desired results, we still insisted on the diagnosis of infection rather than expanding the scope in time to find other possibilities and did specific examinations, which also subjected the patient to undergo several unnecessary operations.

In addition, there is a controversy over the diagnostic criteria for PJI, which also increses the difficulty of diagnosis in this case. Fever, knee swelling and pain were observed in our patient, however, these symptoms lack specificity and are unstable, and only signs of deep tissue involvement (i.e., sinus tracts, effusions, abscesses and widespread necrosis) are the most specific of all PJI-related clinical manifestations (specificity of 97–100%, positive predictive value of 100% and accuracy of 84.3%) [11], which was not present in our patient. Due to the low incidence of PJI and the lack of studies with high quality and grade of evidence, there is still a lack of gold standard for the diagnosis [12]. According to the expert consensus presented at the Second International Consensus Meeting (ICM) on Musculoskeletal Infection in 2018, the main criterion for PJI diagnosis is to culture the same microorganism twice using standard methods. Although synovial grinding tissue fluid was taken for culture, we only had once at each operation, which was not sufficient to diagnose PJI. According to the secondary criteria, the patient met the leukocyte count > 10,000/uL and neutrophils > 90% in synovial fluid, and the single culture was positive, with a cumulative score of 7, which exceeded the 6 required for diagnosis. So in the early and middle stages, we treated it as PJI, but the symptoms didn’t resolved. In addition, although the intraoperative synovial fluid showed yellowish-green turbid suppurative appearance, the determination of suppuration is based on the surgeon’s subjective judgment, and the presence of suppuration or turbid synovial fluid has been reported in both uninfected normal joints and after arthroplasty in the literature [13–15], therefore, suppuration is not a basis for the diagnosis of PJI.

Throughout the diagnostic and treatment process, there seem to be reasonable explanations for our misdiagnosis at the time. However, if we do not overlook subtle differences from previous performance, it can lead us in the right direction.

At last, we summarized some take-home clinical messages of this case as lessons:

- AOSD can highly indicated by high fever, rash, joint symptom, sore throat, increased white blood cell count, negative blood culture, and failure of antibiotic treatment while the first three symptoms are essential for its diagnosis.
- When the patient’s symptoms, physical examinations and examination results are not corresponding to the initial diagnosis, the scope of screening should be expanded, and the early negative results should be reviewed in time.
- The clinical manifestations of many diseases vary greatly. Patients can go to unrelated departments due to different first symptoms. Therefore, in order to reduce misdiagnosis, it is important to have a complete medical history inquiry, conduct comprehensive physical examinations and corresponding laboratory examinations, stay alert of subtle changes in the course of the disease, and finally make a thorough analysis.

## Data Availability

The datasets used and analysed during the current study are available from the corresponding author on reasonable request.

## Abbreviations

AOSD: Adult-onset Still’s disease
CRP: C-reactive protein
ESR: Erythrocyte sedimentation rate
PJI: Prosthetic joint infection
TKA: Total knee arthroplasty (TKA)
DAIR: Debridement, antibiotics, implant retention (DAIR)
